# Correspondence

**Published:** 1883-01

**Authors:** 


### CORRESPONDENCE.

To the Editor of The Journal of Comparative Medicine and Surgery:

Mr. Wortman has recently made important discoveries in the bone-beds of the West, and has furnished me with a summary of the facts, which are of interest to the anatomist. In regard to the discovery of Phenacodus and Meniscotherium, constituting the new order *Condylarthra*, he says:

"Prior to the discovery of these skeletons no characters had been found among the Ungulata which indicate a group connecting the Perissodactyla with the elephants and hyrax. But it is now necessary to create a new order, which Prof. Cope designates the *Condylarthra.* The characters on which this division reposes are found in the carpus and the astragalus (hock or ankle bone) and their manner of articulation. The *Perissodactyla* are distinguished by the fact that the scaphoid articulates with two bones below, and the astragalus articulates inferiorly by two nearly flat facets with the cuboid and navicular bones. They are divisible into ten families, including forty-eight genera, variously distributed throughout geologic time; but as only four of these families concern us for the present, I will spare the memory of the reader by not discussing the classification of the others. The first to which attention may be directed is the *Lophiodontidæ* embracing eight well defined genera, which are not positively known to have existed later than the upper Eocene epoch. It may be recognized (1) by the possession of four toes on the anterior and three on the posterior limbs; (2) by the molar and premolar teeth being different; (3) by the non-separation of the anterior and posterior external cusps of the superior molars by an external, rib-like pillar. The next family is is the *Chalicotheriidæ*, to which ten genera are referred. The digital formula is the same as in the *Lophiodontidæ*, as is also the relation of the molar and premolar teeth. The only distinction is found in the separation of the anterior and posterior external lobes by a vertical ridge. The remains of this family range from the lower Eocene to the middle Miocene. The third family is the *Paleotheriidæ*, having three toes on each foot. The molars and premolars are alike, and the inferior molars possess perfect double crescents. The fourth family is the *Equidæ* in which the digital formula is reduced to one toe on each foot. The molars and premolars are alike and highly complex in structure. It is to this family that all the existing horses belong, and it has been traced as far back as the upper Miocene strata. The *Condylarthra*, on the other hand, are effectually separated from the *Perissodactyla* by the non-alternating positions of the carpals and by the possession of an astragalus whose distal face is convex in every direction, as in the carnivora, and unites with the navicular alone. These families are the *Phenacodontidæ*

and *Meniscotheriidæ* whose remains have been found so far only in the lower Eocene deposits of this country. It is interesting to note that they are the most generalized of any known Perissodactyla and supply a link long sought in the evolution of the later and more specialized forms of this order.

Mr. Wortman's discoveries have necessitated a few changes in the genealogy of the horse, which is now as follows :

PERISSODACTYLA - { Equus, Protohippus, Anchippus, Anchitherium, Mesohippus, Lambdotherium, Hyracotherium, Systemodon. — Equus, Hippotherium, Paloplotherium,

AMBLYPODA, Hyodonta (Cope).

CONDYLARTHRA - { Meniscotherium, Phenacodus.

Yours respectfully,

W. H. CLARKE.

NEW YORK CITY.

---

*To the Editor of the Journal of Comparative Medicine and Surgery:*

### THE SOILING SYSTEM *versus* PASTURING FOR SICK AND LAME HORSES.

So far as I know little has appeared in the periodicals or veterinary publications on the above subject. The many important advantages of of the soiling system for sick and lame horses make it worthy the consideration of veterinarians and all who are interested in the welfare of the horse.

Much has been written on the soiling system, but mostly in regard to feeding farm stock in general when in a state of health. My object is to show that, in many cases, this is a more desirable system of treatment for invalid horses than pasturing. It is the custom to send sick and lame horses, and especially those suffering from having been overdriven, into the country to pasture. This has had, and still has, many strong advocates, and although most of the advantages claimed for pasturing are true ; yet, that this is the most desirable thing to do with such animals is a doubtful question, at least it is one that calls for investigation.

Horses taken from warm stables, especially if accustomed to being clothed, and turned out to roam at large not unfrequently catch cold, sometimes ending in severe maladies. They are exposed to the inclemency of the weather, the chilling storm and the scorching sun—and are frequently fatigued and tortured by insects, when traveling over a large extent of country in order to get a sufficient quantity of food, not overlooking the liability to accident, which is very great. It is of common occurrence for horses sent from a large city like New York to fare very poorly. A great deal of the available land surrounding the city is not rich enough to produce good grasses, and even if the ground be fertile there is a long period of extremely hot weather when most of the grass is literally burned up and the unfortunate animals that are turned out on such a waste surely cannot improve very rapidly.

The good effects of pasturing can, I think, be artificially produced and at the same time many of the disadvantages overcome by adopting the soiling system, or in other words putting the invalid horses in paddocks, (inclosures of half an acre would be more serviceable than smaller ones.) They would have as wholesome air and as much of it, as under any other circumstances, and such paddocks would allow of the invalid horses taking sufficient exercise, while they would not be fatigued or strained in obtaining nourishment. They would get the benefit of the dews to which so much has been attributed and in large paddocks the animals would have sufficient liberty to permit of them getting as good effects as if in the best pasture.

In paddocks they could be supplied with a great variety of food, and consequently frequent change of the same. The variety of green food which might be given, is one of the most important advantages of the soiling system. A succession of crops should be grown and the variety as extensive as practicable. Occasionally a little grass should be mown and given in connection with the various other green food that is fed. The proper food as well as medicine is very important, whether the object be to remove inflammation from the legs and feet, improve the general health, or to relieve animals suffering from various other ailments.

One acre of rich ground will produce more food, whatever the crop may be, than a dozen acres of the non-productive land that is often used for pasturing, and the quality as well as the quantity will be greatly increased. If a great variety of crops is grown and in rotation, so that in turn, from early spring to late in the fall, green fodder may be fed it will be beneficial. Rye that is sown in the Fall will be ready to cut in the Spring long before the pasture is good. Then again all through July and August when the grass is "burned up" various kinds of green fodder will be in perfection and will be greatly relished by the animals. The soiling system, if properly conducted, will be found quite as economical a method of treating invalid horses near great cities, as pasturing. When soiled, the patients are under constant observation, and are at hand to receive such medical treatment as may be necessary. The liability to accidents, as already stated, is much less than when at pasturage.

More *rest* is obtained. It must not be forgotten that rest is the principal factor in the treatment of many ailments to which man and beast are subject.

Respectfully,

WILLIAM HERBERT LOWE.

---

*To the Editor of the Journal of Comparative Medicine and Surgery:*

DEAR SIR—Can you direct me where to find a good article or work which treats of that peculiar disease known as "milk sickness" in cattle, etc.

JACKSON, MO., Nov. 26, 1882.

The literature on the above subject is very meagre. Very little is known concerning its etiology, pathology or treatment.

It seems to be an endemic disease peculiar to the valley of the Mississippi. It occurs at all seasons of the year and derives its name from the fact that it is often communicated to other animals through the milk of animals, and it is also equally well propagated by the flesh.

If any of our readers have a personal knowledge of this singular malady we respectfully request them to write up the subject for the next number of the JOURNAL.—[ED.

